# Aspirin does not modify cardiovascular event risk in endometriosis in the California Teachers Study

**DOI:** 10.1093/ehjopen/oeaf023

**Published:** 2025-05-14

**Authors:** Alison Seitz, Cenai Zhang, Leslie Bull, Hooman Kamel, Halina White, Babak B Navi, Ja Hyun Shin, Jill Berkin, Jed H Kaiser, Vanessa Liao, Ava L Liberman

**Affiliations:** Department of Neurology, University of Washington, Harborview Medical Center, 325 9th Ave, Box #359775, Seattle, WA 98104, USA; Clinical and Translational Neuroscience Unit, Department of Neurology, Feil Family Brain and Mind Research Institute, Weill Cornell Medicine, 520 East 70th Street, Starr 607, New York, NY 10021, USA; University of California Los Angeles David Geffen School of Medicine, 885 Tiverton Dr., Los Angeles, CA 90095, USA; Clinical and Translational Neuroscience Unit, Department of Neurology, Feil Family Brain and Mind Research Institute, Weill Cornell Medicine, 520 East 70th Street, Starr 607, New York, NY 10021, USA; Clinical and Translational Neuroscience Unit, Department of Neurology, Feil Family Brain and Mind Research Institute, Weill Cornell Medicine, 520 East 70th Street, Starr 607, New York, NY 10021, USA; Clinical and Translational Neuroscience Unit, Department of Neurology, Feil Family Brain and Mind Research Institute, Weill Cornell Medicine, 520 East 70th Street, Starr 607, New York, NY 10021, USA; Department of Obstetrics and Gynecology, Weill Cornell Medicine, 525 East 68th Street, Suite J-130, New York, NY 10065, USA; Department of Obstetrics, Gynecology and Reproductive Sciences, Mount Sinai Hospital, 5 E 98th St 2nd Fl, New York, NY 10029, USA; Clinical and Translational Neuroscience Unit, Department of Neurology, Feil Family Brain and Mind Research Institute, Weill Cornell Medicine, 520 East 70th Street, Starr 607, New York, NY 10021, USA; Clinical and Translational Neuroscience Unit, Department of Neurology, Feil Family Brain and Mind Research Institute, Weill Cornell Medicine, 520 East 70th Street, Starr 607, New York, NY 10021, USA; Clinical and Translational Neuroscience Unit, Department of Neurology, Feil Family Brain and Mind Research Institute, Weill Cornell Medicine, 520 East 70th Street, Starr 607, New York, NY 10021, USA

**Keywords:** Endometriosis, Cardiovascular disease, Stroke, Myocardial infarction, Heart disease

## Abstract

**Aims:**

Endometriosis frequently affects reproductive aged females and is associated with increased cardiovascular disease risk. The aims of this study were (i) to confirm the relationship between cardiovascular disease and endometriosis and (ii) to test whether aspirin modified the effect of endometriosis on cardiovascular disease risk.

**Methods and results:**

A longitudinal cohort study was conducted using data from the California Teachers Study from enrolment (1995–1996) through the current administrative end follow-up (31 December 2020). Primary outcome was any incident major adverse cardiovascular event (MACE) defined using validated ICD-9/ICD-10 codes for stroke, myocardial infarction, and coronary heart disease. Inverse probability (IP) weights were used to estimate the causal effect of self-reported endometriosis on cardiovascular events. Of the included 120 435 participants, 13 754 (11.4%) reported history of endometriosis. There were 2159 admissions for MACE in the endometriosis group vs. 16 632 in the non-endometriosis group. After controlling for demographics and vascular comorbidities, risk of MACE was higher in the endometriosis group than in the non-endometriosis group [IP-weighted hazard ratio (HR) 1.10, confidence interval (CI) 1.04–1.15], particularly in participants < 40 years of age (IP-weighted HR 1.48, CI 1.08–2.02). Aspirin use did not modify the effect of endometriosis on MACE (*P* interaction = 0.467). Among participants taking aspirin, the adjusted HR for endometriosis was 1.07 (95% CI, 0.96–1.19) whereas among participants not taking aspirin, adjusted HR was 1.10 (95% CI, 1.04–1.17).

**Conclusion:**

In a large American cohort, endometriosis was associated with increased risk of adverse cardiovascular events, especially in younger participants. Aspirin did not modify this risk. Research to determine how to best reduce cardiovascular risk in endometriosis is warranted.

## Graphical Abstract

**Graphical Abstract:**
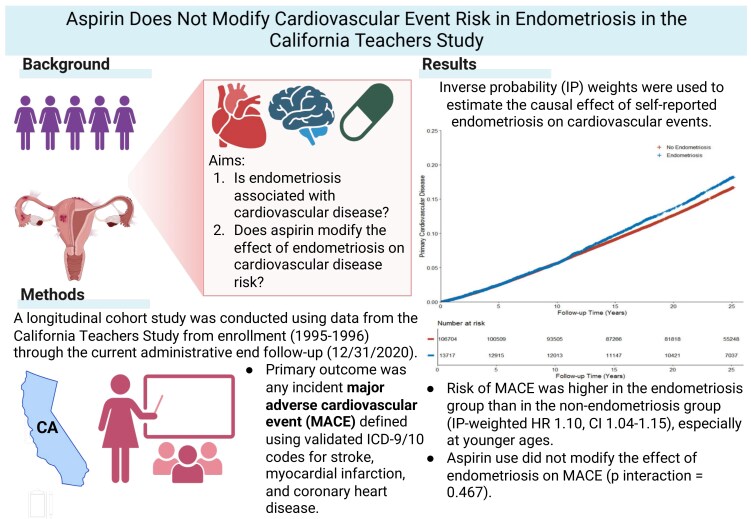

**Editorial for this article: Eur Heart J Open 2025; https://doi.org/10.1093/ehjopen/oeaf044**

## Introduction

Endometriosis is an inflammatory chronic condition that affects approximately 10% of reproductive aged female individuals.^1^ Previous research has suggested that individuals with endometriosis, particularly at younger ages, are at increased risk of hypertension,^2,3^ coronary heart disease,^4–8^ and stroke.^5,7–11^ Alterations in the endogenous inflammatory, immunologic, and hormonal milieu of individuals with endometriosis are unique potential sources of increased cardiovascular disease risk.^12–15^

Despite researchers noting plausible associations between endometriosis and adverse cardiovascular outcomes,^2–7,9–11^ the role of medical therapy for primary prevention in endometriosis has been under-explored. To our knowledge, there is only a single Taiwanese study evaluating the effect of aspirin and statin use on cardiovascular outcomes in females with endometriosis.^16^ The American Heart Association recognized this urgent need in its ‘2024 Guideline for the Primary Prevention of Stroke’ and specifically called for future research examining cardiovascular risk assessment and prevention strategies in those with endometriosis, especially young individuals.^17^

We therefore aimed to (i) confirm the relationship between cardiovascular disease, especially at younger ages, and endometriosis in a large cohort of female teachers and administrators living in the USA^18^ and (ii) evaluate whether or not the use of aspirin modified the effect of endometriosis on risk of adverse cardiovascular events using a large cohort of female patients.

## Materials and methods

### Design and population

We conducted a longitudinal cohort study using data from the California Teachers Study (CTS) which recruited participants in 1995 and 1996 by mailing questionnaires to all active and recently retired female members of the California State Teachers Retirement System. Participants who returned a self-administered questionnaire (*n* = 133 479, median age at enrolment 53 years, range 22–104 years) have been followed prospectively since then, with five subsequent self-administered questionnaires. Additional details regarding CTS have been previously described.^18,19^

For the current study, all participants who completed the baseline CTS questionnaire were eligible for inclusion from their time of enrolment in CTS to the current administrative end of follow-up (31 December 2020). The CTS participants who reported a previous heart attack or stroke on their baseline CTS questionnaire or had a hospitalization for myocardial infarction, coronary heart disease, or any stroke prior to their baseline questionnaire completion date were excluded from our final study cohort.

### Standard protocols and approvals

Institutional Review Board approval was granted through Weill Cornell Medicine as well as the City of Hope National Medical Center to use CTS. Data and statistical code used may be made available to other investigators upon their request to the CTS. We followed the Strengthening the Reporting of Observational Studies in Epidemiology (STROBE) guidelines.^20^

### Exposure

The study exposure was any self-reported history of endometriosis on the baseline questionnaire (1995–1999) or questionnaire four (2005–2008). These two questionnaires are the only ones that asked if a participant has ever had endometriosis. A recent analysis of four international cohorts found that self-report of endometriosis on survey questionnaires could be confirmed by reviewing a combination of surgical, clinical, and pathological records in 84% of cases.^21^ We report the number of CTS participants who answered that they had endometriosis on the baseline questionnaire and on questionnaire four. We considered participants who reported endometriosis on questionnaire four but not on the baseline questionnaire as having endometriosis throughout the study period because most patients with endometriosis have the onset of symptoms in adolescence and it is considered a disease of the entire reproductive lifespan in females despite significant diagnostic delay in many cases.^22^

### Covariates

Study covariates were age, race–ethnicity, and vascular risk factors. Included risk factors were hypertension, diabetes mellitus, current or prior tobacco use, alcohol consumption, obesity (defined as baseline body mass index of ≥30 kg/m^2^), migraine, cancer, and anaemia (defined as needing a blood transfusion).^10,23–25^ We included migraine because migraine with aura has been associated with increased risk of stroke and adverse cardiovascular events, particularly in young women.^26,27^ All covariates were self-reported and selected *a priori*.

### Aspirin covariate

We considered participants regular aspirin users if they reported taking aspirin at least three times weekly for at least 1 year, as in a prior study using the CTS.^28^ To avoid immortal time bias,^29^ we used only the baseline questionnaire (1995–1999) to determine aspirin use. Only aspirin use before major adverse cardiovascular event (MACE) diagnosis was considered. We detail the number of participants who reported taking aspirin on the baseline questionnaire but stopped by questionnaire four.

### Primary outcomes

Our primary study outcome was incident, first-ever MACE. In patients with multiple events during observation, only the first event was counted. The CTS uses California hospital records via the California Office of Statewide Health Planning and Development hospitalization discharge database through probabilistic record linkage based on Social Security number, date of birth, sex, and race–ethnicity to determine outcome events.^19^ For this study, we defined incident of MACE as a first hospital admission resulting in a discharge diagnosis of myocardial infarction (410*, I21*, I22*), coronary heart disease (411*, 412, 413*, 4140*, 4142–4149, V4581, V4582, I20*, I237, I240-I252, I255, I256, I25750, I2575*, I25811, I2582-I2589, I259, Z951, Z955, Z9861), and stroke (430, 431, 433 × 1, 434 × 1, 436, I60, I61, I63, I64) in any position.^30^ All diagnostic codes for MACE have been previously validated (ICD-9: stroke,^31–33^ myocardial infarction, and coronary heart disease^34,35^; ICD-10: stroke,^32,36^ myocardial infarction, and coronary heart disease^37,38^).

### Secondary outcomes

Our secondary study outcomes were (i) ischaemic and haemorrhagic stroke, (ii) coronary heart disease, and (iii) myocardial infarction defined using the aforementioned ICD-9/ICD-10 codes.

### Subgroup analysis: statin covariate

To explore the relationship between statins and cardiovascular risk in endometriosis, we conducted a subgroup analysis of only those participants who completed both the baseline and fourth questionnaire. Questionnaires one through three did not ask about statin use. We considered participants regular statin users if they reported using a statin daily on questionnaire four. Only statin use before MACE diagnosis was considered.

### Subgroup analysis: aspirin within endometriosis subgroups

To explore the relationship between aspirin and cardiovascular risk within participants with and without endometriosis, we completed an additional subgroup analysis using aspirin as the exposure variable within the endometriosis subgroup and then within the non-endometriosis subgroup.

### Statistical analysis

We used standard descriptive statistics to detail baseline characteristics of our study cohort. We reported the number of all MACE as well as the incidence of these events in 100 person-years. Inverse probability (IP) weights were used to estimate the causal effect of self-reported endometriosis history at baseline on the diagnosis of MACE. The utilization of IP weights facilitated the analysis of observational data in a manner akin to a randomized trial with no dropout or loss to follow-up. This IP-weighted methodology enabled us to obtain marginal Kaplan–Meier estimates while accounting for measured confounders.^39,40^

We first calculated the probability of each individual in the cohort having endometriosis, conditioning on their covariate status through logistic regression. For each participant, we took the inverse of the probability obtained from the logistic regression, referred to as the IP weight. These weights were then applied to each individual in the analysis. By weighting individuals by the inverse of their probability of having endometriosis, we gave more weight to underrepresented individuals and less weight to those over-represented, thereby creating a pseudo-population where endometriosis was not associated with the covariates, effectively eliminating measured confounding. We also employed an IP-weighting Cox model to account for censoring, including deaths. The weights were calculated based on the probability of being censored, incorporating baseline characteristics and endometriosis status. By applying these weights in the model, we effectively adjusted for potential bias introduced by censoring events, such as mortality. Cox proportional hazards regression was then fit by weighting participants according to their IP weights to estimate the hazard ratio (HR) and survival curves, with endometriosis as the sole exposure.

The starting point for the primary and secondary outcomes was enrolment, with patients being censored upon death, permanent relocation out of California, or voluntary withdrawal from the CTS. For the subgroup analysis of statin use, the starting point was the completion of questionnaire four, with participants being censored in the same manner as mentioned previously.

We completed the same analysis stratified by age at baseline survey completion using age groups: age < 40, age 40–54, and age ≥ 55 consistent with prior literature on endometriosis and cardiovascular risk.^4^ These age cut-offs are clinically salient because the definition of cardiovascular disease in the young is from age 18 through 39^41^ and the fact that most women undergo menopause by age 55.^42,43^ We then stratified by regular aspirin use and statin use, regardless of age, to examine whether regular aspirin and statin use acted as effect modifiers for endometriosis and cardiovascular risk. Covariates included in the models were age at baseline, race–ethnicity, and vascular risk factors. We used a complete case assessment and did not account for missing data because all participants included in the CTS answered the question for our main variable of interest, endometriosis history, in the baseline questionnaire. The threshold for statistical significance was set as ≤0.05. Statistical analyses and plots were completed in R (version 4.0.2) and completed by Cenai Zhang at Weill Cornell Medicine in New York.^44^

## Results

### Participant characteristics

**Figure 1:**
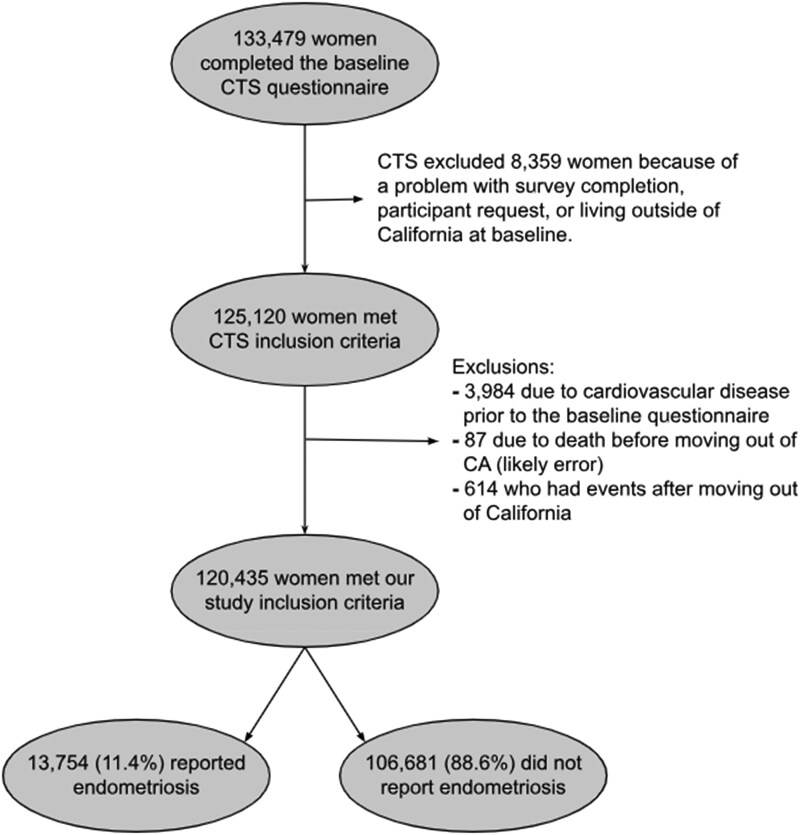
Inclusion flowchart. Inclusions and exclusions of participants from the California Teachers Study.

We included a total of 120 435 participants in our final study cohort (*Figure 1*). Of these participants, 13 754 (11.4%) reported a history of endometriosis. Compared to participants without endometriosis, those with endometriosis were slightly younger (mean age 53.0 vs. 52.3) and much more likely to have a history of migraine (18.3% vs. 28.7%) and severe anaemia (12.5% vs. 17.4%). Participants with endometriosis were also more likely to report regular use of aspirin (10.5% vs. 12.6%), statin (23.4% vs. 26%), anti-hypertensives (12% vs. 13.7%), and oral contraception (65.5% vs. 74.2%) (*Table 1*). Among the 13 754 participants who reported having endometriosis, 10 874 (79%) indicated this diagnosis on the baseline questionnaire and an additional 2880 (20.9%) reported it on questionnaire four. Of the 12 898 participants that reported taking aspirin on the baseline questionnaire, 5546 (43.0%) completed questionnaire four with only 686 of participants reporting that they had stopped taking aspirin on questionnaire four.

**Table 1:** Demographics

	No endometriosis (*n* = 106 681)	Endometriosis (*n* = 13 754)
Demographics		
Mean age at baseline, years (standard deviation)	53.0 (14.6)	52.3 (11.5)
Race–ethnicity, *n* (%)		
Non-Hispanic White	92 090 (86.3%)	12 047 (87.6%)
Hispanic	4671 (4.4%)	541 (3.9%)
Asian	3066 (2.9%)	414 (3.0%)
Non-Hispanic other	3008 (2.8%)	372 (2.7%)
Non-Hispanic Black	2896 (2.7%)	276 (2.0%)
Unknown	950 (0.9%)	104 (0.8%)
Socioeconomic quartile *n* (%)^a^		
1: low	4692 (4.5%)	536 (4.0%)
2	18 338 (17.5%)	2231 (16.5%)
3	34 483 (32.9%)	4373 (32.3%)
4: high	47 362 (45.2%)	6380 (47.2%)
Vascular risk factors		
Obesity, *n* (%)	14 057 (13.8%)	1929 (14.5%)
Former tobacco use, *n* (%)	29 946 (28.3%)	4099 (30.0%)
Current tobacco use, *n* (%)	5387 (5.1%)	639 (4.7%)
Any alcohol use, *n* (%)	66 798 (66.3%)	8843 (67.0%)
Diabetes, *n* (%)	2709 (2.5%)	358 (2.6%)
Hypertension, *n* (%)	17 052 (16.0%)	2484 (18.1%)
Migraine, *n* (%)	19 569 (18.3%)	3946 (28.7%)
Cancer, *n* (%)	9990 (9.4%)	1397 (10.2%)
Severe anaemia^b^, *n* (%)	12 840 (12.5%)	2313 (17.4%)
Medications		
Regular aspirin use^c^, *n* (%)	11 159 (10.5%)	1739 (12.6%)
Oral contraceptives, *n* (%)	67 403 (65.5%)	9826 (74.2%)
Statin, *n* (%)	13 190 (23.4%)	2379 (26.0%)
Anti-hypertensive, *n* (%)	12 850 (12.0%)	1890 (13.7%)

Demographic information, comorbidities, and medications for included participants with and without endometriosis.

^a^Defined by CTS from 1990 census block group variables (occupation, education, and income).

^b^Defined as needing a blood transfusion.

^c^Participants were classified as regular aspirin users if they reported taking aspirin at least three times weekly for at least 1 year on the baseline questionnaire.

### Primary outcomes

**Figure 2:**
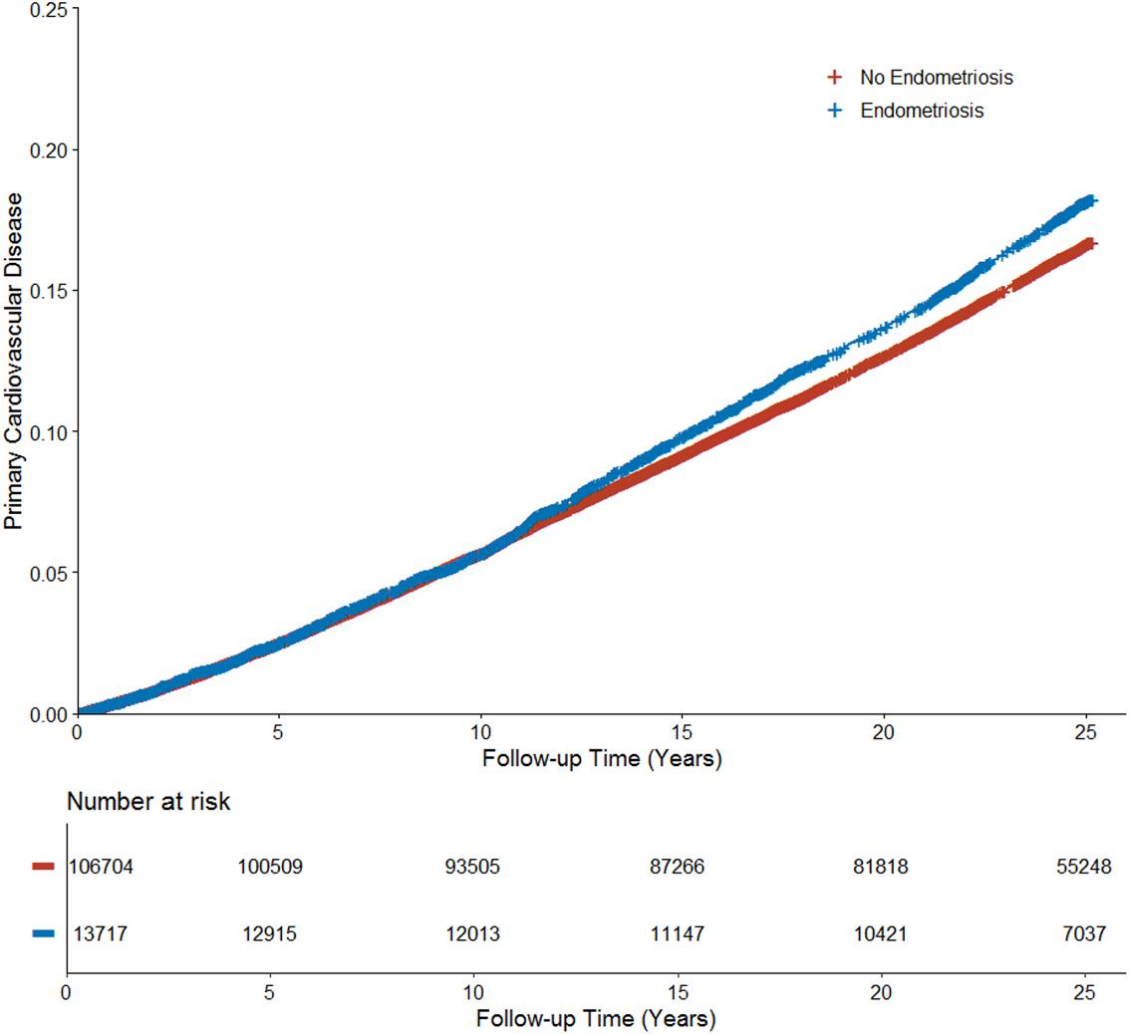
Cardiovascular events in no endometriosis vs. endometriosis. Inverse probability–weighted Kaplan–Meier curves estimating cumulative incidence functions of major adverse cardiovascular events for no endometriosis vs. endometriosis accounting for age at baseline, race–ethnicity, and all self-reported vascular risk factors.

The median follow-up time was 25 years [interquartile range (IQR), 21.4–25.2] in participants with endometriosis and 25 years (IQR, 21.5–25.1) in participants without endometriosis. The incidence rate of MACE was 0.74 [95% confidence interval (CI), 0.71–0.77] per 100 person-years among participants with endometriosis vs. 0.73 (95% CI, 0.72–0.75) per 100 person-years among participants without endometriosis. After adjusting for demographics, confounding, and dropouts, endometriosis was associated with an increased risk of MACE (IP-weighted HR, 1.10; 95% CI, 1.04–1.15; *Figure 2*).

In our age-stratified analysis, we found that endometriosis was most strongly associated with an elevated risk of MACE among participants under 40 years old (IP-weighted HR, 1.48; 95% CI, 1.08–2.02). The incidence rate of MACE was 0.14 (95% CI, 0.11–0.19) per 100 person-years for participants with endometriosis, compared to 0.08 (95% CI, 0.07–0.09) per 100 person-years for those without it in this age group (*Table 2*).

**Table 2:** Cardiovascular event rates in no endometriosis vs. endometriosis

	Endometriosis (*n* = 13 754)		No endometriosis (*n* = 106 681)	
	*n* (%)	Per 100 person-years	*n* (%)	Per 100 person-years
Total events	2159 (15.7%)	0.74 (0.71–0.77)	16 632 (10.9%)	0.73 (0.72–0.75)
Taking aspirin	435 (25.0%)	1.26 (1.14–1.38)	3053 (27.4%)	1.40 (1.35–1.45)
Not taking aspirin	1724 (14.3%)	0.67 (0.64–0.70)	13579 (14.2%)	0.66 (0.65–0.67)
Age				
<40	52 (3.2%)	0.14 (0.11–0.19)	345 (1.8%)	0.08 (0.07–0.09)
40–55	580 (8.4%)	0.38 (0.35–0.41)	2949 (7.0%)	0.31 (0.30–0.32)
≥55	1527 (29.3%)	1.48 (1.41–1.56)	13338 (29.8%)	1.53 (1.50–1.56)

Rates of major adverse cardiovascular events by endometriosis, aspirin use, and age group, unweighted.

For participants aged 40–54 years, the incidence rate of MACE was 0.38 (95% CI, 0.35–0.41) per 100 person-years among those with endometriosis, vs. 0.31 (95% CI, 0.30–0.32) per 100 person-years among those without it. The IP-weighted HR was 1.16 (95% CI, 1.06–1.27).

**Figure 3:**
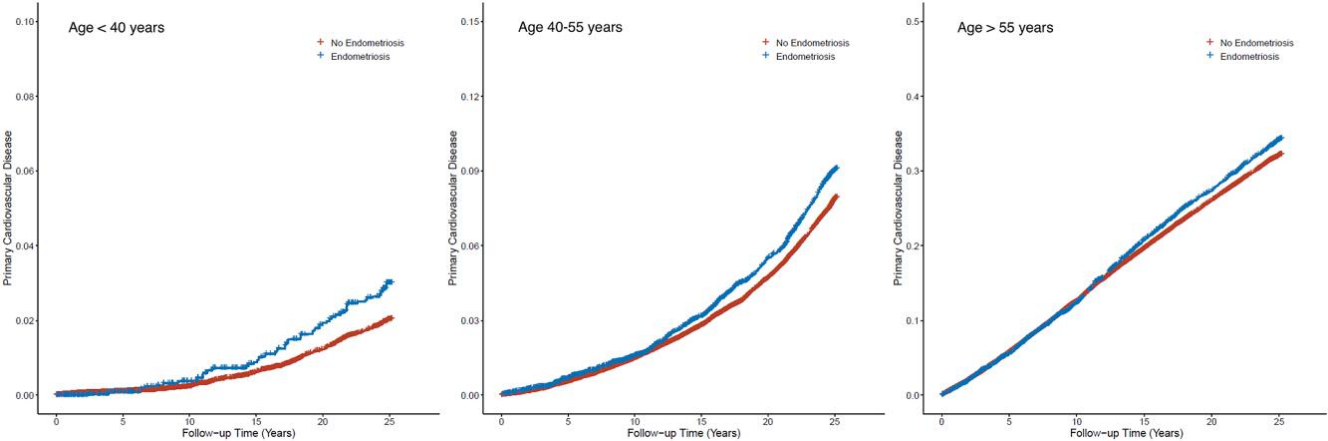
Cardiovascular events in no endometriosis vs. endometriosis by age group. Inverse probability–weighted Kaplan–Meier curves estimating cumulative incidence functions of major adverse cardiovascular events for no endometriosis vs. endometriosis by age group accounting for race–ethnicity and all self-reported vascular risk factors.

Among participants aged 55 years or older, the incidence rate of MACE was 1.48 (95% CI, 1.41–1.56) per 100 person-years for participants with endometriosis, compared to 1.53 (95% CI, 1.50–1.56) per 100 person-years for those without. Although the crude incidence was lower in the endometriosis group, following adjustment for confounding and dropouts using IP-weighting, the IP-weighted HR was 1.07 (95% CI, 1.01–1.13), indicating that endometriosis was associated with an increased risk of MACE in this age group as well (*Figure 3*).

The incidence rate of MACE among participants taking aspirin (*n* = 12 898) was 1.38 (95% CI, 1.34–1.43) per 100 person-years, in contrast to participants not taking aspirin, who had a rate of 0.66 (0.65–0.67) per 100 person-years (see Supplementary material online, *Table S1*). Among participants taking aspirin, the incidence rate of MACE was 1.26 (95% CI, 1.14–1.38) per 100 person-years for participants with endometriosis, compared to 1.40 (95% CI, 1.35–1.45) per 100 person-years for those without (IP-weighted HR, 1.07; 95% CI, 0.96–1.19). Among participants not taking aspirin, the incidence rate of MACE was 0.67 (95% CI, 0.64–0.70) per 100 person-years for participants with endometriosis, compared to 0.66 (95% CI, 0.65–0.67) per 100 person-years for those without (IP-weighted HR, 1.10; 95% CI, 1.04–1.17). The *P* value for the interaction of aspirin use and endometriosis was 0.467.

### Secondary outcomes

The risk of stroke and coronary heart disease was similarly increased in the endometriosis group compared to the non-endometriosis group (stroke: IP-weighted HR, 1.11; 95% CI, 1.02–1.21; coronary heart disease: IP-weighted HR, 1.12; 95% CI, 1.06–1.19). However, for myocardial infarction, there was no difference in risk between the endometriosis and non-endometriosis groups (adjusted HR, 1.04; 95% CI, 0.94–1.16).

### Subgroup analyses

Out of 59 480 participants who completed both the baseline and fourth questionnaires and had no MACE before questionnaire four, 13 127 (22.8%) reported daily statin use. The median follow-up time was 15 years (IQR, 14.3–15.1). Among participants taking statin, the incidence rate of MACE was 1.52 (95% CI, 1.37–1.67) per 100 person-years for participants with endometriosis, compared to 1.55 (95% CI, 1.49–1.62) per 100 person-years for those without (IP-weighted HR, 1.10; 95% CI, 0.98–1.23). Among participants not taking statin, the incidence rate of MACE was 0.80 (95% CI, 0.74–0.86) per 100 person-years for participants with endometriosis, compared to 0.80 (95% CI, 0.77–0.82) per 100 person-years for those without (IP-weighted HR, 1.06; 95% CI, 0.97–1.15). The *P* value for the interaction of statin use and endometriosis was 0.866.

In the endometriosis subgroup, aspirin use at baseline was associated with increased risk of MACE (IP-weighted HR 1.25; 95% CI, 1.11–1.41). In the non-endometriosis subgroup, aspirin use at baseline was also associated with increased risk of MACE (IP-weighted HR 1.24; 95% CI, 1.18–1.30).

### Comment

In our study of 120 435 female teachers and administrators in the CTS, those with endometriosis had a higher risk of MACE, especially at younger ages, than those without endometriosis. We found that aspirin did not modify the effect of endometriosis on cardiovascular disease risk. To our knowledge, this is one of the first studies to assess aspirin as a potential modifier of MACE risk in patients with endometriosis.

Multiple prior studies have shown that the risk of various adverse cardiovascular events is elevated in patients with endometriosis.^4–6,8–10,16,45^ The HR point estimate for MACE in patients with endometriosis in our study was similar to the estimated hazard for cardiovascular disease in three studies,^5,8,9,45^ lower than the estimated hazard for coronary heart disease in two studies,^6,16^ and lower than the estimated hazard for stroke in one study (see Supplementary material online, *Table S2*).^10^

Our finding that an increased MACE risk associated with endometriosis was highest before age 40 is also consistent with extant data, though the absolute risk is low. Three previous studies showed a higher risk of coronary heart disease at younger ages in patients with endometriosis.^4,6,16^ Unlike our study, two of these studies found that by their late 50s, there was no difference in risk of coronary heart disease between individuals with vs. without endometriosis.^4,16^ While one study found no impact of age on the association between endometriosis and stroke, that cohort included younger individuals (mean age of 36) than our cohort.^10^ Endometriosis often, but not always, recedes in menopause,^46,47^ presumably leading to a decrease in the association between endometriosis and cardiovascular disease in older individuals. It is also possible that cardiometabolic changes experienced during menopause offset endometriosis-related risk.^48^

A variety of mechanistic pathways have been postulated to explain the association between endometriosis and cardiovascular disease. To begin with, patients with endometriosis have cytokine and pro-angiogenic factor release leading to systemic inflammation.^49–52^ This systemic inflammation can result in endothelial dysfunction which predisposes to arterial atherosclerotic changes in patients with endometriosis.^53–57^ Endometriosis-related pain and stress^58^ are also plausibly related to cardiovascular disease via increased inflammation and sympathetic activity.^59,60^ Additionally, since genetic variants associated with endometriosis^61,62^ overlap with some genetic variants associated with atherosclerosis resulting in cardiovascular disease,^11,63^ the observed association between endometriosis and cardiovascular disease may be partially attributed to shared vascular risk factors causing both conditions. However, some vascular risk factors, like dyslipidaemia^53,54,64^ and smoking,^15,65^ have mixed evidence for association with endometriosis. Finally, endometriosis treatments^66,67^ may increase risk of cardiovascular disease,^4,9,10^ though some data indicate that treatment lowers cardiovascular disease risk^5,16^ or find no difference in risk.^6,16^

We found that neither aspirin nor statin use is a modifier of the effect of endometriosis on MACE in this cohort. In fact, we found that baseline aspirin use was associated with a higher risk of cardiovascular disease across the cohort regardless of endometriosis. In a prior study, aspirin use was associated with a higher risk of coronary heart disease in individuals with endometriosis though the authors did not account for differences in baseline indications for aspirin use, such as history of stroke, between groups.^16^ One potential reason for our finding is that aspirin use may be associated with severe endometriosis which itself may be associated with substantial risk of cardiovascular disease; aspirin can be used as an analgesic for endometriosis, so patients with more severe endometriosis-related pain may be more likely to use aspirin.^4^ Another possibility is that individuals who are regular aspirin users simply have more cardiovascular disease risk from unmeasured confounders (e.g. family history of myocardial infarction at a young age) which we did not account for. One limitation of our study is a lack of information about indication and duration of aspirin use. Finally, given the unique pathway(s) through which endometriosis increases cardiovascular risk discussed above, aspirin may simply not be an effective method of primary prevention in patients with endometriosis.

Our findings regarding statin use should be interpreted cautiously because questions regarding baseline use were prone to survivorship bias. We also do not know the intensity of statins prescribed. In a previous study using national data from Taiwan, statin use was indeed associated with a decrease in coronary heart disease in patients with endometriosis.^16^ Thus, based on our analytical limitations, previous literature, and the known anti-inflammatory properties of statins,^68^ the role of these medications in cardiovascular risk reduction among patients with endometriosis is an important area for future investigation.

Additional study limitations include the fact that we were unable to determine the effects of various endometriosis treatments (e.g. non-steroidal anti-inflammatory medications, oral contraceptives, hormonal suppression, and surgical treatments) on cardiovascular risk nor how many participants with endometriosis receive treatment for this disease. We also do not know whether or not survey participants stayed on various medical treatments, including aspirin and statins, in between surveys. Another study limitation is the over-representation of non-Hispanic White participants who were all teachers or administrators in the CTS which may limit generalizability to the broader population in the USA. As our study relied on observational data, we were unable to account for residual confounding by unmeasured covariates. Major strengths of our study include our use of a large data set which links survey data including information on medication use with hospitalization data, robust outcome ascertainment using validated diagnostic codes, and long follow-up time allowing us to include a substantial number of elderly patients in our analysis.

## Conclusion

In a large cohort of American female teachers and administrators, we confirmed that risk of MACE was increased among those with endometriosis after adjusting for demographics and vascular risk factors. Regular aspirin use did not modify the effect of endometriosis on MACE.

## Lead author biography


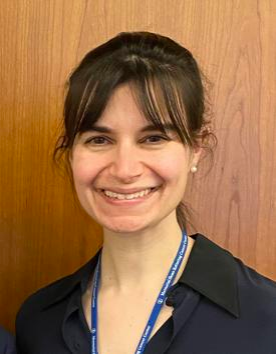


Alison Seitz, MD is an Assistant Professor at University of Washington in Seattle. She graduated from University of California San Francisco School of Medicine. She completed Internal Medicine internship, Neurology residency, and Vascular Neurology fellowship at New York Presbyterian Hospital - Weill Cornell in New York. Dr Seitz's clinical and research interests include sex-specific and sex-predominant risk factors for cerebrovascular disease such as endometriosis, adverse pregnancy outcomes, and migraine.

## Supplementary Material

oeaf023_Supplementary_Data

## Data Availability

Data and statistical code used may be made available to other investigators upon their request to the CTS.
