# Supplementary material for: Aspirin does not modify cardiovascular event risk in endometriosis in the California Teachers Study
Source: Eur Heart J Open. 2025 May 14;5(3):oeaf023. doi: 10.1093/ehjopen/oeaf023 (PMC12076410; doi:10.1093/ehjopen/oeaf023)
Supplement: oeaf023_Supplementary_Data [file oeaf023_supplementary_data.docx]

**Supplemental Appendix**

Page 1 Supplemental Table 1: Major Adverse Cardiovascular Events by Age Unweighted

Page 2 Supplemental Table 2: Studies of the Association of Endometriosis with Cardiovascular Diseases

Supplemental Table 1:

Major Adverse Cardiovascular Events by Age Unweighted

|  | **Aspirin**  **(n=12,898)** | | **No aspirin**  **(n = 107,537)** | |
| --- | --- | --- | --- | --- |
|  | n (%) | Per 100 person-years | n (%) | Per 100 person-years |
| **Total events** | 3488 (27.0%) | 1.38 (1.34-1.43) | 15303 (14.2%) | 0.66 (0.65-0.67) |
| **Age** |  |  |  |  |
| <40 | 18 (2.9%) | 0.13 (0.08-0.21) | 379 (1.8%) | 0.08 (0.07-0.09) |
| 40-55 | 429 (11.1%) | 0.51 (0.46-0.56) | 3100 (6.8%) | 0.30 (0.29-0.31) |
| ≥55 | 3041 (36.1%) | 1.97 (1.90-2.04) | 11824 (28.5%) | 1.44 (1.42-1.47) |

Supplemental Table 2:

Studies of the Association of Endometriosis with Cardiovascular Diseases

| **Dataset** | **Study** | **Primary Outcome** | **Age at study entry (years)** | **Follow up time (years)** | **Adjusted hazard ratio of endometriosis** |
| --- | --- | --- | --- | --- | --- |
| Nurses Health Study II | Mu, 2016^1^ | Myocardial infarction and coronary heart disease | Endometriosis: mean 36 (SD 4); No endometriosis: mean 35 (SD 5) | Up to 20 | 1.62 |
|  | Farland, 2022^2^ | Stroke (ischemic and hemorrhagic) | Endometriosis: mean 36 (SD 4); No endometriosis: mean 35 (SD 5) | Up to 28 | 1.34 |
| Taiwan National Health Insurance | Chiang, 2021^3^ | Myocardial infarction, heart failure, and stroke (ischemic and hemorrhagic) | Median 38 (IQR 31-44) | Mean 9.3 | 1.17 |
|  | Wei, 2021^4^ | Myocardial infarction and coronary heart disease | Mean 38 (standard deviation 8) | Up to 13 | 1.52 |
|  | Li, 2021^5^ | Coronary heart disease | Mean 37 (SD 9) | Mean 7.4 | 1.34 |
| UK Health Improvement Network database | Okoth, 2021^6^ | Ischemic heart disease, heart failure, and cerebrovascular disease | Median 37 (IQR 31-42) | Up to 23 | 1.24 |
| Ontario Health Insurance Plan | Blom, 2023^7^ | Acute myocardial infarction, heart failure, coronary heart disease, and cerebrovascular disease | Mean 36 (SD 8) | Up to 22 | 1.14 |
| Danish nationwide registries | Havers-Borgersen, 2024^8^ | Acute myocardial infarction and ischemic stroke | Median 37.3 (IQR 14.7) | Median 16.1 years | 1.15 |

SD standard deviation; IQR interquartile range.

References

1. Mu F, Rich-Edwards J, Rimm EB, Spiegelman D, Missmer SA. Endometriosis and Risk of Coronary Heart Disease. *Circulation: Cardiovascular Quality and Outcomes*. 2016;9:257–264.

2. Farland LV, Degnan WJ, Bell ML, et al. Laparoscopically Confirmed Endometriosis and Risk of Incident Stroke: A Prospective Cohort Study. *Stroke*. 2022:10.1161/STROKEAHA.122.039250.

3. Chiang H-J, Lan K-C, Yang Y-H, et al. Risk of major adverse cardiovascular and cerebrovascular events in Taiwanese women with endometriosis. *Journal of the Formosan Medical Association*. 2021;120:327–336.

4. Wei C-H, Chang R, Wan YH, Hung Y-M, Wei JC-C. Endometriosis and New-Onset Coronary Artery Disease in Taiwan: A Nationwide Population-Based Study. *Frontiers in Medicine*. 2021;8.

5. Li P-C, Yang Y-C, Wang J-H, Lin S-Z, Ding D-C. Endometriosis Is Associated with an Increased Risk of Coronary Artery Disease in Asian Women. *Journal of Clinical Medicine*. 2021;10:4173.

6. Okoth K, Wang J, Zemedikun D, Thomas G, Nirantharakumar K, Adderley N. Risk of cardiovascular outcomes among women with endometriosis in the United Kingdom: a retrospective matched cohort study. *BJOG: An International Journal of Obstetrics & Gynaecology*. 2021;128:1598–1609.

7. Blom JN, Velez MP, McClintock C, et al. Endometriosis and cardiovascular disease: a population-based cohort study. *Canadian Medical Association Open Access Journal*. 2023;11:E227–E236.

8. Havers-Borgersen E, Hartwell D, Ekelund C, et al. Endometriosis and Long-Term Cardiovascular Risk: A Nationwide Danish study. *European Heart Journal*. 2024:ehae563.
